# Instability of Alien Chromosome Introgressions in Wheat Associated with Improper Positioning in the Nucleus

**DOI:** 10.3390/ijms20061448

**Published:** 2019-03-22

**Authors:** Kateřina Perničková, Veronika Koláčková, Adam J. Lukaszewski, Chaolan Fan, Jan Vrána, Martin Duchoslav, Glyn Jenkins, Dylan Phillips, Olga Šamajová, Michaela Sedlářová, Jozef Šamaj, Jaroslav Doležel, David Kopecký

**Affiliations:** 1 Institute of Experimental Botany, Centre of the Region Haná for Biotechnological and Agricultural Research, Šlechtitelů 31, 78371 Olomouc, Czech Republic; pernickova@ueb.cas.cz (K.P.); kolackova@ueb.cas.cz (V.K.); vrana@ueb.cas.cz (J.V.); dolezel@ueb.cas.cz (J.D.); 2 Department of Botany and Plant Sciences, University of California, Riverside, CA 92521, USA; adam.lukaszewski@ucr.edu (A.J.L.); chaolanf@ucr.edu (C.F.); 3 Department of Botany, Faculty of Science, Palacký University Olomouc, Šlechtitelů 27, 783 71 Olomouc, Czech Republic; martin.duchoslav@upol.cz (M.D.); michaela.sedlarova@upol.cz (M.S.); 4 Institute of Biological, Environmental and Rural Sciences, Aberystwyth University, Aberystwyth, Ceredigion, Wales SY23 3DA, UK; gmj@aber.ac.uk (G.J.); dwp@aber.ac.uk (D.P.); 5 Department of Cell Biology, Centre of the Region Haná for Biotechnological and Agricultural Research, Faculty of Science, Palacký University Olomouc, Šlechtitelů 27, 783 71 Olomouc, Czech Republic; olga.samajova@upol.cz (O.Š.); jozef.samaj@upol.cz (J.Š.)

**Keywords:** chromatin, 3D-FISH, nucleus, introgression, rye, hybrid, wheat, genome stability

## Abstract

Alien introgressions introduce beneficial alleles into existing crops and hence, are widely used in plant breeding. Generally, introgressed alien chromosomes show reduced meiotic pairing relative to the host genome, and may be eliminated over generations. Reduced pairing appears to result from a failure of some telomeres of alien chromosomes to incorporate into the leptotene bouquet at the onset of meiosis, thereby preventing chiasmate pairing. In this study, we analysed somatic nuclei of rye introgressions in wheat using 3D-FISH and found that while introgressed rye chromosomes or chromosome arms occupied discrete positions in the Rabl’s orientation similar to chromosomes of the wheat host, their telomeres frequently occupied positions away from the nuclear periphery. The frequencies of such abnormal telomere positioning were similar to the frequencies of out-of-bouquet telomere positioning at leptotene, and of pairing failure at metaphase I. This study indicates that improper positioning of alien chromosomes that leads to reduced pairing is not a strictly meiotic event but rather a consequence of a more systemic problem. Improper positioning in the nuclei probably impacts the ability of introgressed chromosomes to migrate into the telomere bouquet at the onset of meiosis, preventing synapsis and chiasma establishment, and leading to their gradual elimination over generations.

## 1. Introduction

During interphase, chromosomes are decondensed and occupy distinct regions of the nucleus, called chromosome domains or chromosome territories [1,2]. In flowering plants, chromosome territories show two predominant configurations, known as the Rabl’s orientation and Rosette [3,4] with the former being more frequent. The Rabl’s configuration [5] reflects the orientation of chromosomes from the preceding anaphase: all centromeres are grouped at or close to the nuclear periphery at one pole of the nucleus while telomeres are dispersed toward the opposite pole [6]. Such an orientation presumably simplifies homologue search and the initiation of chromosome pairing in early meiosis. Chromosome pairing is initiated during leptotene (or telomere) bouquet, where all telomeres are located close to each other at one pole of the nucleus. The association of the telomeres into the bouquet at the onset of the first meiotic division facilitates the initiation of synapsis of homologous chromosomes, which in turn is a prerequisite for crossing over and chiasmate pairing at metaphase I [7], both of which are critical for the success of meiosis. It has been clearly demonstrated that misalignment of telomeres of homologues during the bouquet formation restricts synapsis and drastically reduces metaphase I pairing [8,9]. 

Many crops are allopolyploids, that is, products of wide hybridization, a natural process commandeered frequently by plant breeders to widen the gene pool of a crop by introduction of agronomically important alleles. Such introgressions may take the form of new amphiploids, such as triticale (X Triticosecale Wittmack), introgressions of single chromosomes, chromosome arms or even smaller chromosome segments [10]. One of the most successful intergeneric introgressions is the 1RS.1BL centric wheat-rye translocation in wheat (*Triticum aestivum* L.), where the short arm of wheat chromosome 1B is replaced by its counterpart from the rye genome (*Secale cereale* L.). Many other introgressions have been released in wheat breeding and research programs involving rye, barley (*Hordeum* sp.), *Aegilops*, *Agropyron* and *Haynaldia* species [11,12]. 

As a general rule, early generations of interspecific hybrids suffer irregular chromosome pairing which may affect different genomes in various ways. This hampers wider commercial utilization of amphiploids and introgression lines in agriculture. Nishiyama [13] described reduction of chromosome number in successive generations of synthetic decaploid interspecific hybrids of oats (*Avena*) from 2n = 70 to 2n = 42−58. Similarly, reversion to bread wheat (loss of the entire rye genome) has been reported in octoploid triticale (amphiploids of bread wheat with rye) [14]. In tetraploid and hexaploid triticales, rye chromosomes fail to pair more frequently than wheat chromosomes [15]. Among disomic additions and substitutions of individual rye chromosomes in bread wheat, Orellana et al. [16] found reduced metaphase I pairing of rye chromosomes and significantly higher numbers of univalents compared to wheat chromosomes. 

The problem of reduced chromosome pairing in amphiploids has been a subject of much debate over the decades. In wheat-rye hybrids, five different concepts have been presented, but none has been proven to be correct (reviewed in [17]). After several recent studies, a relationship between the behaviour of telomeres and the success of chromosome pairing has gained credence. Murphy and Bass [18] have shown that the desynaptic (*dy*) mutant of maize displays multiple defects in telomere-nuclear envelope interactions, homologous chromosome synapsis, recombination and chromosome segregation. Similarly, Naranjo [19] reported that reduced pairing of rye chromosomes in wheat appears to be a consequence of disturbed migration of rye telomeres into the leptotene bouquet. Telomere positioning and migration are preferentially studied in meiosis. However, our previous study suggested that the problem may be systemic in nature, and aberrant arrangement of telomeres in pollen mother cells (PMCs) is only an extension of their erratic behaviour in other (somatic) tissues. We observed that the frequency of out-of-bouquet rye telomere position at leptotene was virtually identical to that in the nuclei of somatic cells, and was similar to the frequency of synapsis of the normal and inverted chromosome arms in a heterozygote for an inversion of a rye chromosome arm in wheat [20]. 

In this study, we analysed wheat lines with introgressed chromatin from rye, involving disomic whole chromosome substitutions, ditelosomic line and centric (whole arm) translocations in the 3D space of wheat nuclear volume of somatic tissue in order further investigate the possible link between telomere positioning in somatic tissues, chromosome pairing and stability of introgressed alien chromatin. Special attention was paid to the distribution of the telomeres and centromeres. 

## 2. Results

### 2.1. Morphometrical Characteristics of G1 Interphase Nuclei of Wheat-Rye Introgression Lines

**Figure 1:**
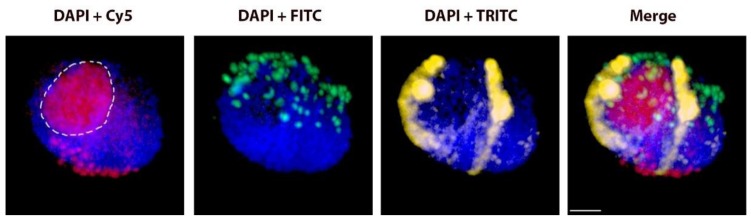
Rabl’s orientation of rye chromosomes. Nucleus with a pair of homologous rye _del_1RS.1RL chromosomes in the proper Rabl’s orientation. Total genomic DNA of rye was labelled with TRITC using Nick translation (yellow color), centromeres of both wheat and rye chromosomes were visualized using oligonucleotide probe (red color), and telomere-specific sequence was PCR-labelled with FITC (green color). Nuclear DNA was counterstained with DAPI (blue color). Note the difference in signal intensity along the chromosome arms with subtelomeric heterochromatin labelled dark yellow and the remaining rye arm labelled light yellow. Nucleoli are indicated by white dashed lines. Scale bar 5 µm.

In total, we analysed 315 nuclei with most of the parameters described in Material and Methods. The morphology of G1 nuclei flow sorted into a polyacrylamide gel ranged from spherical and ellipsoidal to irregular shapes with varying degrees of contortion. Only nuclei with spherical and slightly ellipsoidal shapes were selected for analyses. Nuclear volumes ranged from 753 to 2996 µm^3^ (mean ± SD 1677 ± 506 µm^3^). 3D-GISH analysis showed that the chromosome territories (CTs) of rye chromosome arms appeared as compact structures of regular shapes spanning the entire nucleus and arranged in a typical Rabl’s orientation (Figure 1). Centromeres were generally close to each other and located on the nuclear periphery at one pole, while telomeres were at the periphery of the opposite pole. In a majority of the analysed nuclei, centromeres of rye chromosome arms were closer to each other than their telomeres (Table 1).

### 2.2. Positions of Rye Telomeres and Centromeres Relative to the Nuclear Envelope and Positioning of Rye Telomeres in the Telomere Cluster

In the majority of nuclei, telomeres and centromeres of rye chromosomes/chromosome arms were positioned at the nuclear periphery (NP). As we did not perform any tests for the attachment of telomeres to the inner nuclear envelope, we cannot be certain that the positioning of telomeres or centromeres at the nuclear periphery reflects their attachment to the nuclear envelope. However, it is a safe assumption that the positioning of a telomere away from the nuclear periphery indicates an absence of such attachment. It also needs to be stressed that the position of the nuclear envelope was inferred from the edge of the DAPI-stained chromatin. Therefore, in the absence of definitive proof, all telomeres located away from the edge of nuclear periphery are assumed to be unattached to the nuclear envelope; those at the nuclear periphery are labelled below as “in contact with NP”. 

In a small proportion of nuclei across most of the genotypes, a centromere or a telomere of one rye chromosome arm was positioned away from the NP (Table 2). In control diploid rye, essentially all chromosomes were in Rabl’s orientation with all centromeres positioned at the centromere pole and 99.3% of telomeres located at the telomere pole. Similarly, 94.3% of the telomeres were in contact with the NP. Rye chromosome arms in the introgression lines differed in the proportions of the telomere (log-linear models using likelihood-ratio chi-square test, χ^2^ = 54.36, DF = 12, *p* < 0.001) and centromere (χ^2^ = 25.61, DF = 12, *p* = 0.012) positions relative to the NP, while only minor differences were observed among genotypes in proportions of telomeres positioned at the telomere pole (χ^2^ = 22.08, DF = 12, *p* = 0.036). 

**Figure 2:**
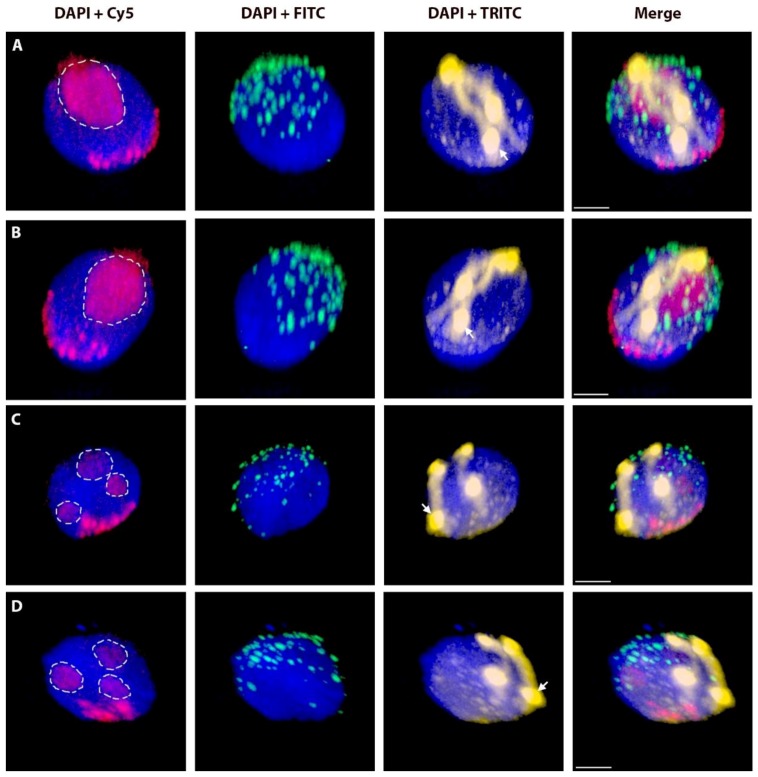
Telomere out-positioning of rye chromosome arms. Two nclei ((**A**,**B**) is the same nucleus from different angle; the same for (**C**,**D**)) with a pair of homologous rye _del_1RS.1RL chromosomes after 3D-FISH. Total genomic DNA of rye was labelled with TRITC using Nick translation (yellow colour), centromeres of both wheat and rye chromosomes were visualized using oligonucleotide probe (red colour), and telomere-specific sequence was PCR-labelled with FITC (green colour). Nuclear DNA was counterstained with DAPI (blue colour). Rye telomeres positioned out of the telomere pole are indicated by arrows. Nucleoli are indicated by white dashed lines. Scale bar 5 µm.

**Figure 3:**
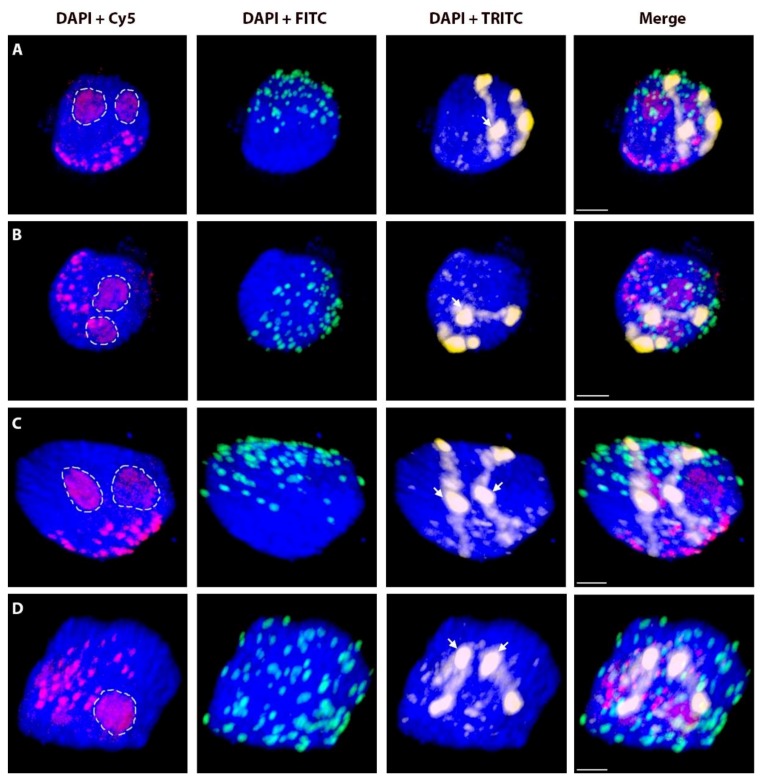
Non-attachment of rye telomeres. Two nuclei ((**A**,**B**) is the same nucleus from different angles; same for (**C**,**D**)) with a pair of homologous rye _del_1RS.1RL chromosomes after 3D-FISH. Total genomic DNA of rye was labelled with TRITC using Nick translation (yellow colour), centromeres of both wheat and rye chromosomes were visualized using oligonucleotide probe (red colour), and telomere-specific sequence was PCR-labelled with FITC (green colour). Nuclear DNA was counterstained with DAPI (blue color). Rye telomeres without visual contact to nuclear envelope are indicated by arrows. Nucleoli are indicated by white dashed lines. Scale bar 5 µm.

**Figure A1:**
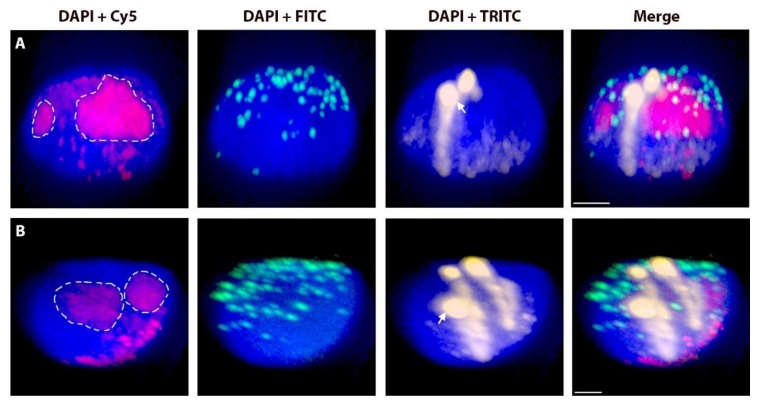
Non-attachment of rye telomeres. Nuclei with a pair of telocentric 1RS rye chromosomes (**A**) and a pair of 1R rye chromosomes (**B**) after 3D-FISH. Total genomic DNA of rye was labelled with TRITC using Nick translation (yellow color), centromeres of both wheat and rye chromosomes were visualized using oligonucleotide probe (red color), and telomere-specific sequence was PCR-labelled with FITC (green color). Nuclear DNA was counterstained with DAPI (blue color). Rye telomeres without visual contact to nuclear envelope are indicated by arrows. Nucleoli are indicated by white dashed lines. Scale bar 5 µm.

**Figure A2:**
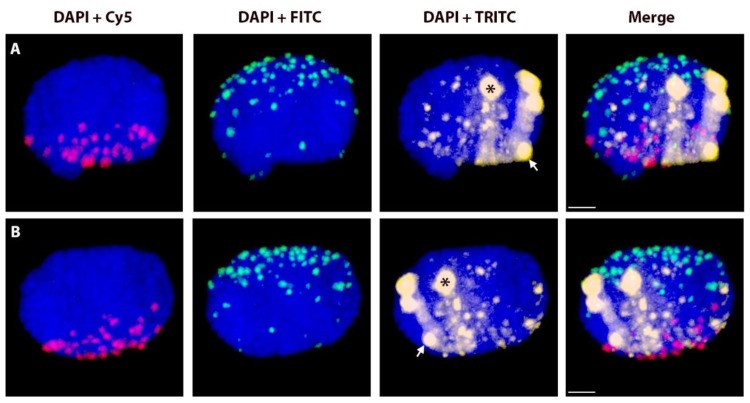
Combination of non-attachment and out-positioning of rye telomeres. A nucleus ((**A**,**B**) is the same nucleus from different angles) with a pair of homologous rye 1RS. _del_1RL chromosomes after 3D-FISH. Total genomic DNA of rye was labelled with TRITC using Nick translation (yellow color), centromeres of both wheat and rye chromosomes were visualized using oligonucleotide probe (red color), and telomere-specific sequence was PCR-labelled with FITC (green color). Nuclear DNA was counterstained with DAPI (blue color). Rye telomere without visual contact to nuclear envelope is indicated by asterisk, rye telomere positioned out of the telomere pole (at the centromere pole) is indicated by arrow. Nucleoli are indicated by white dashed lines. Scale bar 5 µm.

In the majority of cases, centromeres were tightly clustered at one pole of the nucleus; telomeres were located in the about one third of the nuclear volume opposite to the centromere pole (see measurements of C-C and T-T in Table 1). Rarely, a telomere was located away from the telomere pole (Figure 2, Figure A2 and Video S1) and/or away from the nuclear envelope (NE) (Figure 3; Figure A1 and Figure A2 and Video S2). The frequencies of telomere proper positioning and telomere contact with the nuclear periphery (NP) were correlated (Spearman correlation coefficient, r_s_ = 0.57, *p* = 0.043), and they appeared to correlate with the length of a chromosome arm (telomere proper positioning: r_s_ = 0.57, *p* = 0.040, telomere in contact with NP: r_s_ = 0.53, *p* = 0.063): shorter arms were less likely to be in contact with the NP and more frequently were out-of-position (Table 2). Chromosome length and arm ratio had no effect on the proper positioning of telomeres in Rabl’s orientation and the proportions of centromeres and telomeres in contact with the NP. Shortening of 1RS by a deletion reduced the in-contact with the NP frequency from 93.2% (in 1R) to 46.0% (in _del_1RS.1RL) and proper positioning from 100% to 82%. In all cases, telomeres of long arms were in contact with the NP much more frequently than those of the short arms. Even the reduction in length of 1RL did not change this pattern. Telomeres of the 1RS arm were less likely to be in contact with the NP relative to those of the _del_1RL arm (90.5% vs. 97.6%, respectively). Telocentric chromosome t1RS had its telomere at the nuclear periphery with the same frequency as the short arm of 1R (93.2%) and was always properly arranged in the Rabl’s configuration (the same as 1RS in normal 1R). Interestingly, the telomere located at the centromere of t1RS was positioned at the centromere pole but less frequently in contact with the NP than the centromere of 1R (79.5% vs. 90.9%).

### 2.3. Chromosome Pairing and Transmission Rate

**Figure 4:**
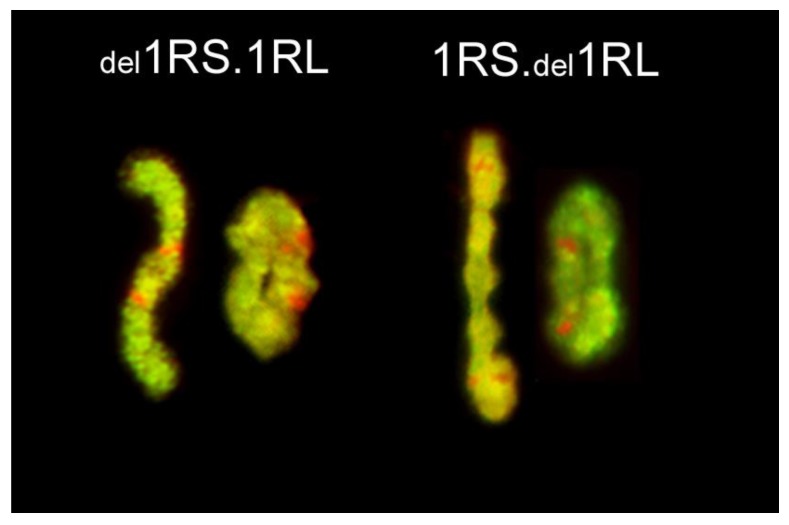
Metaphase I pairing of chromosomes 1R with deletions. From left to right: chromosome _del_1RS.1RL paired as a rod in the short arm and as a ring and chromosome 1RS. _del_1RL paired as a rod in the long arm and as a ring. Short arms are marked by non-hybridized (red) bands of the NOR region.

The pairing frequencies of rye chromosome arms, whether in chromosome 1R or in centric translocations, were high, but varied for different chromosome arms and even for the same arms but in different translocations or configurations. In centric translocations 1RS.1BL and 2BS.2RL, rye chromosome arms paired less frequently than the wheat arms present (90.0% vs. 94.0% and 93.9% vs. 95.9%, respectively). In the disomic substitution and deletion lines of chromosome 1R, the short arm (1RS) always paired less frequently than the long one. Deletion of a large portion of an arm, whether in the short or the long arm, did not have any major effect on pairing frequency of the arm with the deletion, or the other arm of the chromosome (Figure 4).

The transmission rate of rye chromosomes or chromosome arms in wheat-rye centric translocation was also high, but with some variation among the lines (Table 3). Centric translocations were highly stable with the transmission rate in most cases at 100%. The only exception was 5RS.5BL with a transmission rate of 98.6% (one out of 71 plants was nullisomic for this translocation). Similarly, high stability was observed in 1R (99.4%; one monosomic among 80 plants) and _del_1RS.1RL (98.7%; two monosomics among 80 plants). On the other hand, much lower transmission rates were observed for 1RS._del_1RL (91.9%; 13 monosomics among 80 plants) and ditelosomic 1RS (87.2%; two nullisomics and 15 monosomics among 74 plants).

## 3. Discussion

**Figure 5:**
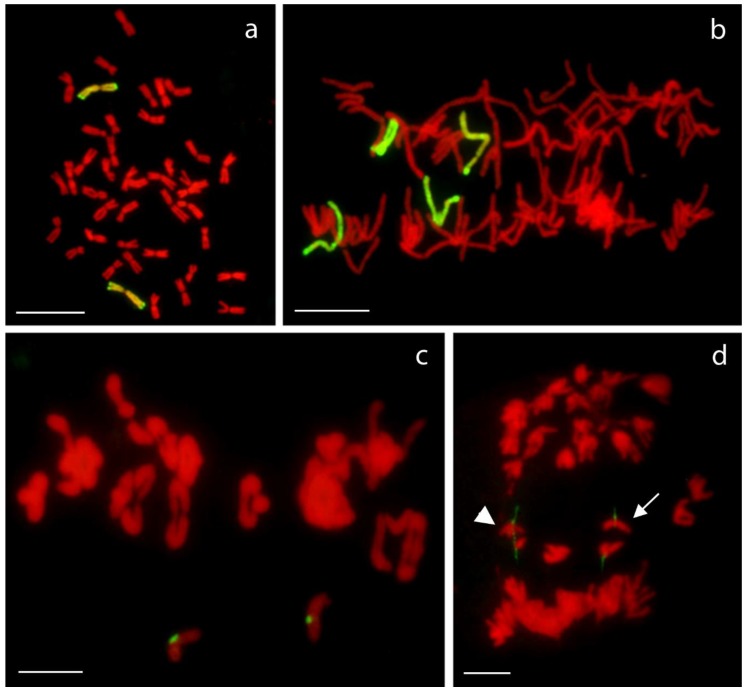
Mitotic (**a**,**b**) and meiotic (**c**,**d**) behavior of rye chromosome pair introgressed into wheat background. Rye chromosomes usually behaves perfectly normal during mitotic metaphase (**a**) and anaphase (**b**), while reduced pairing was observed during meiotic metaphase I (**c**). This may result in the separation of sister chromatids (arrow) or misdivision (in this case one arm of one chromatid is being separated from the rest of the chromosome; arrowhead) during anaphase I (**d**). Rye mitotic chromosomes were visualized using labelled genomic DNA of rye (labelled with FITC; green color), while rye-specific centromeric probe was used for meiotic preparations (labelled with FITC; green color). Scale bar 10 µm.

Interspecific hybrids and allopolyploids frequently display genome instability. This instability may take different forms, such as parental chromosome competition. In *Lolium* × *Festuca* hybrids, the *Lolium* genome predominates: chromosomes of *Festuca* are slowly, but continuously replaced by those of *Lolium* in consecutive generations [26,27]. The mechanism underlying this phenomenon is not yet clear. Alternatively, genome instability may manifest itself as reduced meiotic pairing of one of the parental genomes, causing the higher univalency rate for the low pairing genome, and its gradual elimination from the hybrid. Wheat-rye hybrids are perhaps the best studied in this respect, with known cases of complete elimination of the rye genome, especially from octoploid triticales [14]. This phenomenon is also well documented in single chromosome wheat-rye addition lines [16]; as a general rule rye chromosomes pair poorly and are eliminated over time, which requires careful control of such lines to maintain their status. It has been argued that meiotic instability of wheat-rye hybrids is a consequence of mis-matched chromosome pairing control systems of the two parental species, and, especially, the effect of the *Ph1* system of wheat [17]. However, the *Ph1* system in wheat appears to impose strict stringency levels for crossover formation, and rye homologues in established triticale lines and wheat-rye addition lines are virtually the same. In stocks analysed in this study, rye chromosome arms always paired less frequently than their wheat counterparts. This fits well with the general pattern of rye chromosome behaviour when introgressed into wheat. As shown earlier by [16], rye chromosomes introgressed into wheat as disomic substitution or addition lines generate univalents in frequencies ranging from 1 to 19% (6% on average; Figure 5). Similarly, rye univalents are twice as frequent as those of wheat in tetraploid triticale, despite equal proportions of wheat and rye genomes [15]. 

Naranjo [22] indicated that reduced metaphase I pairing of rye chromosomes in wheat might be a consequence of a lower probability of rye telomeres clustering into the leptotene bouquet at the onset of meiosis. Proper positioning of telomeres in the bouquet is believed to be prerequisite for the initiation of synapsis, and thus, regular chromosome pairing [7]. In our previous work [20], we observed a surprising agreement between the frequency of out-of-position telomeres at leptotene and in somatic nuclei on the one hand, and the failure of metaphase I pairing on the other. However, the situation is probably much more complex. There is chromosome movement at the onset of meiosis mediated by protein complexes of the inner nuclear envelope [28]. Such movement may be less frequent, or perhaps impossible, if the telomeres are not attached to the nuclear envelope (NE) already in the premeiotic interphase. The frequencies of non-attached rye telomeres observed in this study (0–12.5% for normal length chromosome arms) are again surprisingly similar to the frequencies of their out-of-bouquet positioning at the leptotene-zygotene transition as shown by Naranjo [19,22] and the frequencies of their pairing failure ([16] and our results). Interestingly, the transmission rate of rye introgressions in disomic centric wheat-rye translocations was high. This is because wheat chromosome arms in such translocations pair with normal frequencies assuring normal disjunction of the translocated chromosomes, their regular inclusion into the products of meiosis and then normal transmission of rye chromatin into subsequent generations. This is despite reduced (relative to diploid rye) pairing of rye chromosome arms. On the other hand, general instability of ditelosomics (such as t1RS analysed here) appears to be associated with lower frequencies of telomeres attached to the NE (here detected as telomeres in contact with NP). Correlation also exists between the out-positioning of rye telomeres in diploid rye nuclei observed here (0.66%) and average pairing failure per arm in a population of rye (0.21%) [29,30]. Thus, what appears to emerge from rather fragmentary data is that there is a direct link between somatic arrangement of chromosome arms, the leptotene bouquet and metaphase I pairing success. 

The study of Naranjo [19] and our results indicate that there may be a relationship between chromosome arm length and the positioning of that arm’s telomere at the telomere cluster/pole. Naranjo found that only 83.3% and 73.5% of telomeres of the short arms of chromosomes 1R and 6R, respectively, were located in the telomere cluster at the leptotene-zygotene transition, while none of 1RL telomeres and only 0.5% of 6RL telomeres were out of the telomere clusters [19]. Similarly, the lowest frequency of rye telomere clustering was for chromosome arm 5RS, the shortest rye chromosome arm, and the highest observed frequencies were for the arm 6RL, the longest in the karyotype [19,22,31]. This correlates well with our results, where telomeres of shorter chromosome arms were attached to the NE (or positioned at the telomere cluster/pole) less frequently than the telomeres of longer chromosome arms and significantly higher frequencies of pairing of long arms compared to short arms of chromosomes 1R, 2R and 5R in disomic addition lines (84.5 vs. 91.0, 64.8 vs. 96.8% and 31.0 vs. 79.0, respectively) [16]. Similarly, reduction in length of 1RS arm by deletion in the 1R introgression line caused reduction of its telomere attachment from 93.2% (in regular 1R introgression) to 46%. 

## 4. Materials and Methods 

### 4.1. Plant Material 

**Figure 6:**
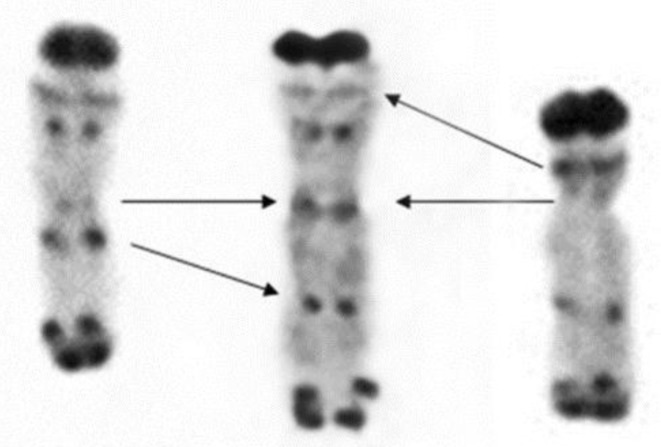
Three 1R chromosomes differing in the length of their arms. From left to right: 1RS._del_1RL with deletion of about 30% of the 1RL arm (proximal part), regular 1R chromosome and _del_1RS.1RL with deletion of about 36% of 1RS arm (proximal part).

The plant material consisted of a set of lines of hexaploid bread wheat (*Triticum aestivum* L., 2n = 6× = 42) cv. ‘Pavon 76’ with disomic (homozygous) introgressions of rye chromosomes or chromosome arms: substitution of rye chromosome 1R for wheat chromosome 1A [1R(1A)], ditelosomic addition line 1RS, a deletion line _del_1RS.1RL where ca. proximal 36% of the short arm is missing, a deletion line 1RS._del_1RL where proximal ca. 30% of the long arm is missing (Figure 6), and centric wheat-rye chromosome translocations 1RS.1BL, 1RS.1DL, 1AS.1RL, 2RS.2BL, 2BS.2RL and 5RS.5BL. The telosomic line and all centric translocation lines were created by centric misdivision of complete normal chromosomes of rye and their wheat homoeologues; the deletion chromosomes were identified during selection of centric translocations [32]. As a control, we used a population of rye (*Secale cereale* L.) cv. Dankowskie Zlote. About 25 seedlings were used for each line. 

Note on terminology: this manuscript uses the original terminology of Bridges for chromosome aberrations where “deficiency” is a loss of a terminal chromosome segment and “deletion” indicates a loss of an intercalary segment [33].

### 4.2. Isolation of Nuclei and Flow Sorting

Seeds of the introgression lines were germinated in Petri dishes on moist filter paper at 25 °C in the dark. Root tips from young seedlings were collected and fixed in 2% (*v*/*v*) freshly prepared formaldehyde in meiocyte buffer A (15 mM PIPES, pH 6.8, 80 mM KCl, 20 mM NaCl, 0.5 mM EGTA, 2 mM EDTA, 0.15 mM spermine tetra-HCl, 0.05 mM spermidine tri-HCl, 1 mM dithiothreitol, 0.32 M sorbitol) for 20 min at 5 °C. After fixation, root tips were washed three times for 15 min at room temperature in meiocyte buffer A. Meristem tissue of root tips was cut and transferred into a 5 mL sample tube containing 400 µL of meiocytes buffer A, and homogenized using a Polytron PT1200 homogenizer (20,000 rpm/13 sec). The homogenate was filtered through 20 µm nylon mesh into a 5 mL polystyrene tube and stored on ice until used. The nuclear suspension was stained with 2 µg/mL DAPI (4’,6-diamidino-2-phenylindole). Nuclei in G1 phase of the cell cycle were identified and sorted using a FACSAria II SORP flow cytometer (BD Biosciences, San Jose, CA, USA) into a sample tube containing 10 µl of meiocyte buffer A [34]. About 50,000 nuclei at G1 were obtained from one sample prepared from 50 root tips. Using flow cytometry enabled us to have a sample of the nuclei exclusively from one phase of cell cycle (G1), rather than a mixture of cells from different phases, which could bias the results from different lines. 

### 4.3. Probe Preparation and 3D-FISH

Total genomic DNA of *S. cereale* L. was labelled with Texas Red or TRITC using a Nick Translation Kit (Roche Applied Science, Penzberg, Germany) according to manufacturer’s instructions, and applied as a probe. Total genomic DNA of rye labelled the rye chromosome arms yellow, and their subtelomeric heterochromatin dark yellow. Both rye and wheat centromeres were visualized by an oligonucleotide probe based on the sequence of clone pHind258 [35] and directly labelled with Cy5. The telomeric probe was prepared using PCR and FITC-directly labelled nucleotides. Total genomic DNA of wheat was sheared to 200–500 bp fragments by boiling and used as blocking DNA at a ratio of 1:150 (probe/blocking DNA). As a control, we used rye nuclei with probes specific for telomeres and centromeres. 

3D-FISH experiments were performed according to [36,37] with minor modifications. The suspension of flow-sorted nuclei was dripped onto a 22 × 22 mm coverslip and mixed with acrylamide solution (30% Acrylamide/Bis-acrylamide Mix Solution, ratio 29:1, Sigma-Aldrich, St. Louis, USA) in meiocyte buffer A, ammonium persulfate (20%) and sodium sulphate anhydrous (20%). The drop of solidifying nuclear suspension was covered with another coverslip and the “sandwich” was placed in an oven at 37 °C for 1 h for polymerization. Thereafter, the coverslip “sandwich” was gently separated by a razor blade. The coverslip with polymerized nuclei was washed in meiocyte buffer A in a small Petri dish for 3 × 5 min at room temperature. The coverslip was then placed on a new slide with a drop of hybridization mixture for FISH. The FISH mixture included probes, blocking DNA, 70% formamide and 2× saline sodium citrate (SSC). Hybridization was conducted according to [37]. The nuclei were counterstained with 1.5 µg/mL DAPI in Vectashield antifade mounting medium (Vector Laboratories, Burlingame, USA).

### 4.4. Image Acquisition and Analysis

Probed nuclei were optically sectioned using an inverted laser spinning disk microscope (Axio Observer Z1, ZEISS) and ZEN Blue 2012 software and an inverted motorized microscope Olympus IX81 equipped with a Fluoview FV1000 confocal system (Olympus, Tokyo, Japan) and FV10-ASW software. Fluorescein (binding to telomere-specific repeats) was excited by a 488 nm line of an argon laser, while Texas Red and TRITC (total rye genomic DNA) were excited by He-Ne laser at 543 nm or 561 nm. Cy5 (labelling both rye and wheat centromeres) was excited at 639 nm. The excitation of DAPI (to visualize nuclear DNA) was performed by a 405 nm diode laser.

For each nucleus, 80–120 optical sections in 160 – 200 nm steps were taken and merged into a 3D model. Subsequent analyses were performed using Imaris 9.2 software (Bitplane, Oxford Instruments, Zurich, Switzerland). Imaris applications ‘Contour Surface’, ‘Spot Detection’ and ‘3D Measurement’ were used for manual analysis of each nucleus. The volume and the centre of the nucleus (CN) were determined from the rendering of primary intensity of DAPI staining using the function ‘Surfaces’. The ‘Spot’ function was used to mark the positions of centromeres (C) and telomeres (T). Distances between the centromeres of rye homologues (C-C) and between their telomeres (T-T) were measured using the ‘Line’ function. 

Special attention was paid to the positioning of rye telomeres and centromeres relative to the nuclear envelope and to the (wheat) telomere cluster. No additional steps were taken to visualize the nuclear envelope: its position was inferred from the edge of the DAPI-stained chromatin. ‘Display Adjustment’ was used to adjust the channel contrast and thus, to improve the visualization of all analysed objects. Between 21 and 40 nuclei were analysed per genotype.

### 4.5. Chromosome Pairing and Transmission Rate 

Meiotic pairing of selected chromosomes was analysed using standard protocols for material collection, fixation and genomic in situ hybridization (GISH) described in detail previously [34]. Briefly, from each sampled flower, a portion of one anther was removed, fresh-squashed in a drop of acetocarmine and if the desired meiotic stage was present, the remaining anthers from the flower were fixed in a mixture of absolute ethyl alcohol and glacial acetic acid in proportion 3:1 at 37 °C for a week and stored at −20 °C until used. Chromosome pairing was analysed on squashed preparations using GISH. Rye chromosomes and chromosome arms were visualized with DIG-labelled total genomic rye DNA and anti-DIG FITC, with wheat DNA sheared to ca. 200–500 bp fragments as a blocking DNA at 1:150 ratio (probe: blocking DNA). Following hybridization, preparations were counterstained with 1.5% propidium iodide (PI) in VectaShield antifade (BioRad, Hercules, CA, USA), mounted and observed under a microscope. Individual arms of rye chromosome 1R were identified by the presence of an unlabelled NOR band on the short arm. Pairing frequencies of individual arms of chromosome 1R were taken from a previous study [25]. The same protocol was used on root-tip meristems to estimate transmission rates to further generations. Seeds were germinated on wet filter paper in Petri dishes, root tips were collected to ice water for 26–30 h and fixed in a mixture of absolute alcohol and glacial acetic acid (3:1) at 37 °C for seven days. GISH was done the same way as for meiotic preparations with a probe prepared from total genomic DNA of rye and unlabelled genomic DNA of wheat as a blocking DNA. 

## 5. Conclusions

To conclude, our study indicates that reduced chromosome pairing of introgressed alien chromosomes (or chromosome arms) may be predetermined already in somatic cells, and is more systemic in nature: telomeres of rye chromosomes in wheat often fail to attach to the nuclear envelope, and less frequently assume proper positions within the telomere cluster. This may hamper their migration to the telomere bouquet at the onset of meiosis resulting in reduced synapsis and reduced metaphase I pairing. Consequently, reduced metaphase I pairing lowers the transmission rate of such chromosomes to successive generations, and thus, destabilizes the integrity of a hybrid genome. 

## Figures and Tables

**Table 1:** Morphometrical characteristics of rye chromosome arms in wheat-rye introgression lines. The values (Mean ± SD) of arm length, distances between centromere to centromere (C-C) and telomere to telomere (T-T) (given in µm) were normalized according to the volume of the nucleus (absolute distance in µm/nuclear volume × 1000).

Introgression	Nuclear Volume (µm^3^)	Arm Length (µm)	C-C (µm)	T-T (µm)
1AS.1RL	1545 ± 224	7.73 ± 1.48	4.01 ± 1.98	4.88 ± 1.71
1RS.1BL	1501 ± 362	8.21 ± 1.69	3.49 ± 1.50	4.18 ± 1.66
1RS.1DL	1998 ± 285	6.86 ± 1.31	2.83 ± 1.15	3.71 ± 1.74
2RS.2BL	1597 ± 490	8.51 ± 2.58	3.38 ± 1.70	4.09 ± 1.95
2BS.2RL	1510 ± 357	8.96 ± 1.62	4.05 ± 1.80	4.99 ± 2.25
5RS.5BL	1656 ± 329	6.95 ± 1.54	3.06 ± 1.29	3.97 ± 1.48
_del_1RS.1RL	1986 ± 390	S ^1^: 4.04 ± 1.03	2.88 ± 1.37	3.86 ± 1.79
		L ^2^: 7.56 ± 1.67		3.56 ± 1.87
1RS. _del_1RL	2006 ± 500	S ^1^: 6.18 ± 1.58	3.57 ± 1.69	4.20 ± 1.92
		L ^2^: 5.21 ± 1.41		4.12 ± 1.53

^1^ S: short arm ^2^ L: long arm.

**Table 2:** Frequencies of proper positioning of telomeres of rye chromosome arms (at the telomere pole) and rye telomeres and centromeres in contact with the nuclear periphery (NP).

Introgression	Rye Chromosome arm Length (Mb) ^3^	Chromosome Length (Mb)	Arm Ratio	Number of Nuclei	Telomere Proper Positioning (%)	Telomere in Contact with the NP (%)	Centromere in Contact with the NP (%)
1AS.1RL	626	902	2.27	25	98.00	98.00	84.00
1RS.1BL	423	959	1.27	40	98.75	98.75	100.00
1RS.1DL	423	804	0.90	40	97.50	95.00	98.75
2RS.2BL	595	1102	0.85	40	98.75	96.25	98.75
2BS.2RL	693	1116	1.64	40	98.75	100.00	100.00
5RS.5BL	346	928	1.68	40	93.75	87.50	96.25
_del_1RS.1RL	short arm: 271 ^1^ long arm: 626	897	2.31	25	1RS: 82.00 1RL: 100.00	1RS: 46.00 1RL: 100.00	90.00
1RS._del_1RL	short arm: 423 long arm: 438 ^2^	861	1.04	21	1RS: 92.90; 1RL: 100.00	1RS: 90.50 1RL: 97.60	88.10
1R(1A)	short arm: 423 long arm: 626	1049	1.48	22	1RS: 100.00 1RL: 100.00	1RS: 93.20; 1RL: 100.00	90.90
t1RS	423	423	-	22	100.00	93.20	79.50

^1^ deletion of about 36% of 1RS arm (proximal part); ^2^ deletion of about 30% of 1RL arm (proximal part); ^3^ estimated values of chromosome and chromosome arm length and arm ratio have been calculated from karyotypes of Schlegel et al. [21] and Naranjo [22] for rye and Gill et al. [23] for wheat and genome size estimations [24].

**Table 3:** Pairing of rye chromosome arms during metaphase I and their transmission to the next generation.

Introgression	Number of PMC	Chromosome Pairing	Number of Progeny	Transmission (%)
1AS.1RL			63	100.0
1RS.1BL	50	1RS: 90.0; 1BL: 94.0	32	100.0
1RS.1DL			30	100.0
2RS.2BL			38	100.0
2BS.2RL	49	2BS: 95.9; 2RL: 93.9	76	100.0
5RS.5BL			71	98.6
_del_1RS.1RL	60	1RS: 90.0; 1RL: 100.0	80	98.7
1RS._del_1RL	59	1RS: 89.8; 1RL: 94.9	80	91.9
1R(1A)	289 ^1^	1RS: 80.8; 1RL: 94.4	80	99.4
t1RS			74	87.2

^1^ [25].

## Supplementary Materials

Supplementary materials can be found at https://www.mdpi.com/1422-0067/20/6/1448/s1. **Video S1 (.mp4)**. Visualization of nucleus of disomic deletion line 1RS._del_1RL after 3D-FISH. Total genomic DNA of rye was labelled with TRITC using Nick translation (yellow color), centromeres of both wheat and rye chromosomes were visualized using oligonucleotide probe (red color), and telomere-specific sequence was PCR-labelled with FITC (green color). Nuclear DNA was counterstained with DAPI (blue color). Out-positioning of one rye chromosome arm is indicated by arrow. **Video S2 (.mp4)**. Visualization of nucleus of disomic deletion line _del_1RS. 1RL after 3D-FISH. Total genomic DNA of rye was labelled with TRITC using Nick translation (yellow color), centromeres of both wheat and rye chromosomes were visualized using oligonucleotide probe (red color), and telomere-specific sequence was PCR-labelled with FITC (green color). Nuclear DNA was counterstained with DAPI (blue color). Non-attachment of telomeres of both rye _del_1RS chromosome arms is indicated by arrows.

## Abbreviations

3D-FISH	Three-dimensional fluorescent in situ hybridization
CN	Centre of the nucleus
CT	Chromosome territory
GISH	Genomic in situ hybridization
NE	Nuclear envelope
NOR	Nucleolar organizing region
NP	Nuclear periphery
PMC	Pollen mother cell
